# BESSiE: a software for linear model BLUP and Bayesian MCMC analysis of large-scale genomic data

**DOI:** 10.1186/s12711-016-0241-x

**Published:** 2016-09-02

**Authors:** Vinzent Boerner, Bruce Tier

**Affiliations:** Animal Genetics and Breeding Unit, University of New England, Armidale, 2351 Australia

## Abstract

**Background:**

The advent of genomic marker data has triggered the development of various Bayesian algorithms for estimation of marker effects, but software packages implementing these algorithms are not readily available, or are limited to a single algorithm, uni-variate analysis or a limited number of factors. Moreover, script based environments like R may not be able to handle large-scale genomic data or exploit model properties which save computing time or memory (RAM).

**Results:**

BESSiE is a software designed for best linear unbiased prediction (BLUP) and Bayesian Markov chain Monte Carlo analysis of linear mixed models allowing for continuous and/or categorical multivariate, repeated and missing observations, various random and fixed factors and large-scale genomic marker data. BESSiE covers the algorithms genomic BLUP, single nucleotide polymorphism (SNP)-BLUP, BayesA, BayesB, BayesC$\pi$ and BayesR for estimating marker effects and/or summarised genomic values. BESSiE is parameter file driven, command line operated and available for Linux environments. BESSiE executable, manual and a collection of examples can be downloaded http://turing.une.edu.au/~agbu-admin/BESSiE/.

**Conclusion:**

BESSiE allows the user to compare several different Bayesian and BLUP algorithms for estimating marker effects from large data sets in complex models with the same software by small alterations in the parameter file. The program has no hard-coded limitations for number of factors, observations or genetic markers.

## Background

In quantitative genetics, various software packages are available for the analysis of phenotypic observations with linear mixed models, which can be categorised by the algorithm used to infer dispersion and location parameters of the modelled factors: (a) restricted maximum likelihood (REML) based software, and (b) Bayesian inference based software using Markov chain Monte Carlo (MCMC) methods (e.g. Gibbs sampling). While various REML software packages specifically designed for quantitative genetics are widely used and well documented, (e.g. ASREML [1], WOMBAT [2], DMU [3], REMLF90 [4], VCE [5]), software packages that apply the MCMC methodology are less common  (e.g. GIBBSF90 and THRGIBBSF90 [4], GS3 [6], BAYESR [7], MCMCglmm [8]). The relatively small number of MCMC software packages for quantitative geneticists may reflect the disadvantage of MCMC methodology in terms of processing time. In addition, large-scale genomic marker data [e.g. single nucleotide polymorphisms (SNPs)] that emerge in the late 2000 can easily be accommodated in existing REML software via approaches such as single marker regression, genomic best linear unbiased prediction (GBLUP) [9] or SNP-BLUP [10]. By contrast, several Bayesian algorithms for sampling dispersion and location parameters of genomic markers have been proposed (e.g. “BayesA”, “BayesB”, “BayesC$\pi$”, “BayesR”  [11–13]), which differ only slightly but require adjustments in the source code, thus making it more difficult to develop and maintain a software package which covers all algorithms.

The aim of this article is to describe the software BESSiE which is designed for uni- and multivariate BLUP and Bayesian analysis of linear mixed models in quantitative genetics allowing for various factors, algorithms, large-scale genomic data and both continuous as well as categorical observations.

## Software description

### Program modes

BESSiE allows for two different modes, “BLUP” and “GIBBS”.

Mode “BLUP” performs a best linear unbiased analysis of the specified linear mixed models given the observed data and supplied variances of random factors. The results are best linear unbiased estimations for levels of fixed factors and best linear unbiased predictions for levels of random factors.

Mode “GIBBS” performs a Bayesian analysis of the specified linear mixed model given the observed data using supplied variances of random factors as starting values or as prior knowledge in an MCMC Gibbs sampling approach, which is expanded by Metropolis–Hasting steps if required. The results for factor levels and for co-variances are draws from their conditional posterior distributions as given in [14]. In addition, for factors that model genetic markers, the results are draws from a posterior distribution as specified in [11] (“BayesA” and “BayesB”), in [12] (“BayesC$\pi$) or in [13] (“BayesR”).

### Models

The super-set model to be fitted in BESSiE may be written as:

$$\begin{aligned} \left( \begin{array}{c} {\mathbf y} _1 \\ . \\ {\mathbf y} _{\text {n}} \\ \end{array} \right)= & \left( \begin{array}{ccc} \mathbf X _1 & . & 0 \\ . & . & . \\ 0 & . & \mathbf X _{\text {n}} \\ \end{array} \right) \left( \begin{array}{c} \mathbf b _1 \\ . \\ \mathbf b _{\text {n}} \\ \end{array} \right) \\&+\left( \begin{array}{ccccccc} \mathbf Z _{1,1} & . & \mathbf Z _{1,\text {k}} & . & 0 & 0 & 0\\ . & . & . & . & . & . & . \\ 0 & 0 & 0 & . & \mathbf Z _{\text {n},1} & . & \mathbf Z _{\text {n,k}} \\ \end{array} \right) \left( \begin{array}{c} \mathbf u _{1,1} \\ . \\ \mathbf u _{\text {n,k}} \\ \end{array} \right) \\&+\left( \begin{array}{ccc} \mathbf Q _1\mathbf M &. & 0 \\ . &. &. \\ 0 &. & \mathbf Q _{\text {n}}\mathbf M \\ \end{array} \right) \left( \begin{array}{c} \mathbf g _1 \\ . \\ \mathbf g _{\text {n}} \\ \end{array} \right) + \left( \begin{array}{c} \mathbf e _1 \\ . \\ \mathbf e _{\text {n}} \\ \end{array} \right) \end{aligned}$$

where $(\mathbf y _1,.,\mathbf y _{\text {n}})'$, $(\mathbf b _1,.,\mathbf b _{\text {n}})'$, $(\mathbf u _{1,1},.,\mathbf u _{\text {n,k}})'$, $(\mathbf g _1,.,\mathbf g _{\text {n}})'$ and $(\mathbf e _1,.,\mathbf e _{\text {n}})'$ are vectors of phenotypic observations of linear or categorical scale (including repeated and/or missing observations), fixed effects, random non-marker effects [$1..\text {k}$] and random marker effects, **X**, **Z** and **Q** are matrices relating the effects to their respective observations, **M** is a matrix of marker genotypes of dimension “number of genotyped individuals” $\times$ “number of markers” and the subscripts are for trait 1 to n. Values in **X** may be dummy variables or linear co-variables, where for the latter the order of polynomial regression is user-defined. Values in $(\mathbf u _1,.,\mathbf u _{\text {n}})$ are assumed to be distributed $N([0,.,0]',\mathbf A \otimes \mathbf {\Sigma })$, $N([0,.,0]',\mathbf G \otimes \mathbf {\Sigma })$, $N([0,.,0]',\mathbf I \otimes \mathbf {\Sigma })$ or $N([0,.,0]',\mathbf K \otimes \mathbf {\Sigma })$, where **A** is the pedigree-derived numerator relationship matrix, **G** is a relationship matrix derived from genetic markers, **I** is an identity matrix, **K** is an unknown, but symmetric and positive definite matrix of dimension “number of factor levels” $\times$ “number of factor levels” provided by the user, and $\mathbf {\Sigma }$ is a co-variance matrix of factors. Note that all random non-marker effects can be fitted together.

Effects of genetic markers $(\mathbf g _1,.,\mathbf g _{\text {n}})'$ can be obtained from “BayesA” and “BayesB” [11], “BayesC$\pi$” [12], “BayesR” [13] or ridge regression SNP BLUP [10]. For “BayesA”, “BayesB” and “BayesC$\pi$”, default values for parameters of the algorithms and prior distributions of marker variances are derived from the related publications, but can also be specified by the user.

If the co-variance structure of a factor is $\mathbf G \otimes \mathbf {\Sigma }$, where **G** is a genomic relationship matrix, **G** can be pre-calculated by the user and read from a file, or can be calculated by the program from a file of genomic markers. In the latter case, two methods are provided to calculate **G** described in [9] and [15], the latter being similar to the second method in [9] except for the diagonal elements.

Residuals are assumed to be distributed $N([0,.,0]',\mathbf I \otimes \mathbf R )$, where **R** is the residual co-variance matrix of dimension $\text {n}\times \text {n}$. However, to account for observations with different residual variances (e.g. de-regressed breeding values), a co-variance $\varvec{\Omega }$ can be modelled, where $\varvec{\Omega }$ is a matrix of diagonal blocks containing $\varvec{\omega }_1\sigma _{{\text {e}}_1}^2$ to $\varvec{\omega }_{\text {n}}\sigma _{{\text {e}}_{\text {n}}}^2$ in the diagonal elements of the diagonal blocks, and $\sqrt{\varvec{\omega }_1\varvec{\omega }_{\text {n}}}\sigma _{e_{1,\text {n}}}$ in the diagonal elements of the off-diagonal block which links trait 1 and trait n, where $\varvec{\omega }_1$ and $\varvec{\omega }_{\text {n}}$ are vectors of weights for trait 1 and n, and $\sigma _{{\text {e}}_1}^2$, $\sigma _{{\text {e}}_{\text {n}}}^2$ and $\sigma _{{\text {e}}_{1,\text {n}}}$ are the residual variances and co-variance of the traits.

In multivariate analysis using “BayesA”, “BayesB”, “BayesC$\pi$” or “BayesR” effects of genetic markers are estimated from

$$\begin{aligned}&\left( \left[ \begin{array}{ccc} \mathbf Q _1\mathbf M &. & 0 \\ . & . & . \\ 0 & . & \mathbf Q _{\text {n}}\mathbf M \\ \end{array} \right] ' \mathbf R ^{-1} \left[ \begin{array}{ccc} \mathbf Q _1\mathbf M & . & 0 \\ . & . & . \\ 0 & . & \mathbf Q _{\text {n}}\mathbf M \\ \end{array} \right] \right. \\&\quad +\left. \left[ \begin{array}{ccc} \varvec{\Sigma }_1 & . & 0 \\ . & . & . \\ 0 & . & \varvec{\Sigma }_{\text {n}} \\ \end{array} \right] ^{-1} \right) \\&\quad \left( \begin{array}{c} \mathbf g _1 \\ . \\ \mathbf g _{\text {n}} \\ \end{array} \right) \\&\quad =\left( \begin{array}{ccc} \mathbf Q _1\mathbf M & . & 0 \\ . & . & . \\ 0 & . & \mathbf Q _{\text {n}}\mathbf M \\ \end{array} \right) ' \mathbf R ^{-1}\\&\left( \left[ \begin{array}{c} \mathbf y _1 \\ . \\ \mathbf y _{\text {n}} \\ \end{array} \right] -\left[ \begin{array}{ccc} \mathbf X _1 & . & 0 \\ . & . & . \\ 0 & . & \mathbf X _{\text {n}} \\ \end{array} \right] \left[ \begin{array}{c} \mathbf b _1 \\ . \\ \mathbf b _{\text {n}} \\ \end{array} \right] \right. \\&\quad -\left. \left[ \begin{array}{ccc} \mathbf Z _{1,1} & . & 0 \\ . & . & . \\ 0 & . & \mathbf Z _{\text {n,k}} \\ \end{array} \right] \left[ \begin{array}{c} \mathbf u _{1,1} \\ . \\ \mathbf u _{\text {n,k}} \\ \end{array} \right] \right) \end{aligned}$$

where $\varvec{\Sigma }_1$ to $\varvec{\Sigma }_{\text {n}}$ are diagonal matrices of dimension “number of markers” $\times$ “number of markers” of which elements contain the marker variances generated according to the Bayesian method specified for trait 1 to n. Thus, the co-variances between the effects of a genetic marker on trait 1 to n are assumed to be zero. The user should be aware that modelling markers in a multivariate analysis that assumes no co-variance between effects of a single marker on different phenotypes may lead to spurious results if a polygenic (pedigree-based) component is not included in the model.

### Program input

**Fig. 1:**
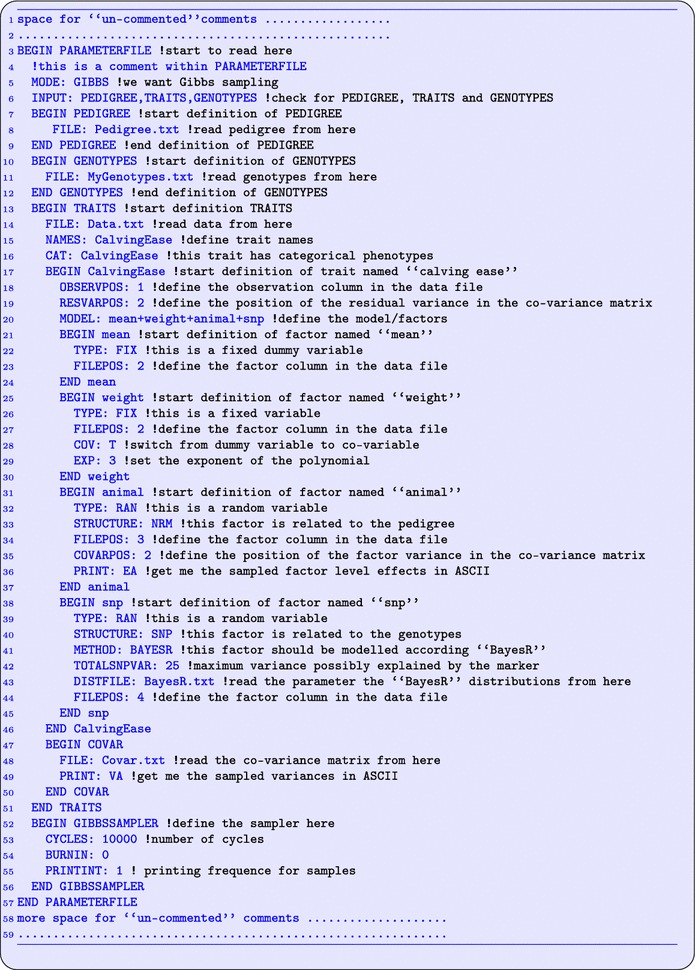
Example parameter file

BESSiE reads all necessary information from a parameter file, which also contains names and locations of other input files. These might be files containing data, genotypes, pedigree, co-variances and other matrices. Program information in the parameter file is identified by case sensitive keywords, and the block structure within these keywords are nested, which ensures robustness against input errors, easy extension for new input parameters, and allows for extensive commenting outside the block structure (see Fig. 1).

BESSiE data files must contain only numeric input and assumes the user will be able to renumber all modelled factors. However, BESSiE conducts an extensive data check and will provide informative error messages in case it detects any inconsistency in factor numbering.

If BESSiE is used to estimate effects of genetic markers via one of the six above mentioned implemented algorithms, it expects that markers are coded “0”, “2” and “1” for both the homozygous and the heterozygous genotype. Thus, missing markers within genotypes are not accommodated for. Markers are provided via an ASCII file containing one line per genotype and as many columns as markers. Thus, genotypes of different markers must not be separated by any character, which allows for a reduced file sizes. However, BESSiE checks for valid marker coding while reading through that file.

### Program output

BESSiE generates various outputs in ASCII or Fortran binary files where the desired output can be chosen by the user. In mode “BLUP”, the output may include solutions for factor levels conditional on the variances provided. In mode “GIBBS”, the output may include draws from conditional posterior distributions of factor levels and factor co-variances. For random marker effects modelled by “BayesA”, “BayesB”, “BayesC$\pi$” or “BayesR”, effects and variances for each marker as well as the total variance explained by genetic markers and summarised genomic values for each genotype can optionally be written in the file. Moreover, for algorithms “BayesC$\pi$” and “BayesR”, the output may include the draws from Beta/Dirichlet conditional posterior distributions assigning probabilities to the distributions from which markers may come from, and marker-to-distribution assignment statistics. By default BESSiE also generates an extensive log file which contains informative messages in case of input errors and information about the current state of the program while a Bayesian analysis is running, for example.

### Technical details

For Bayesian analysis, BESSiE uses a blocked Gibbs sampler as described in [14], but only for fixed and random non-marker effects. That is, random non-marker effects are sampled from their assigned distributions. The right-hand side of the mixed model equation (MME) is corrected for these draws and the MME is subsequently solved. Draws are added back to the related MME solutions and the resulting values can be regarded as draws from the conditional posterior of each random non-marker effect. Effect sizes and variances of genetic markers modelled by “BayesA”, “BayesB”, “BayesC$\pi$” or “BayesR” are obtained from a single site Gibbs sampler using phenotypic observations corrected for fixed and random non-marker effects. For solving the MME in a BLUP analysis and in blocked Gibbs sampling, BESSiE uses a preconditioned gradient solver. This may slow down the number of Gibbs sampler cycles per second if the data set is very large, but it is assumed to accelerate convergence drastically [16, 17]. BESSiE does not set up the coefficient matrix of the MME. While this prevents exploitation of parallel processing, computer memory requirements are kept to a manageable level even for genome-wide association studies including whole-genome sequences and “BLUP” estimations including millions of animals. However, parts of the preconditioned gradient solver steps are parallelised. The algorithm that is used to obtain dispersion and location parameters when trait observations are of categorical scale is described in [18] and [19].

### Speed, memory requirements, implementation and availability

BESSiE has no hard coded limitations in terms of number of traits, factors, genotypes and markers, and has been tested on very large data sets.

As an example, a bi-variate analysis with 4420 individuals genotyped for 510,174 SNPs, 19,549 individuals in the pedigree, seven fixed effects and a polygenic random effect per trait, and SNP effects modelled according to “BayesR” with four distributions required $\sim$4.3 GB of RAM and about seven real time seconds on an Intel(R) Core(TM) i7-3770 processor to sample all location and dispersion parameters once.

Another example is a uni-variate analysis of publicly available mouse data (http://mus.well.ox.ac.uk/mouse/) consisting of 1940 phenotypes and genotypes, where genotypes contained 8516 SNP genotypes, and a model including only the mean and a random marker factor modelled by the “BayesR” algorithm with four distributions. When executed on a notebook with an Intel(R) Core(TM) i7-2637M processor, BESSiE used $\sim$72 MB of RAM and need 5 real time milliseconds to sample all location and dispersion parameters once.

BESSiE is written in Fortran 2008, command line operated, parameter file driven and comes with an extensive manual. It is available for 64bit Unix-like operation systems only. BESSiE uses the Intel Math Kernel library [20] for random number generation and matrix operations and is therefore optimised for Intel architecture. However, it will run on AMD architecture but run time may increase.

BESSiE comes free of charge for the scientific community, but users are required to credit its use in any publication. Commercial users must contact the authors. BESSiE executable, manual and a collection of examples can be downloaded from http://turing.une.edu.au/~agbu-admin/BESSiE/. BESSiE is under ongoing development, and due to the number of features, some combinations of algorithms and/or modelled factors may not have been tested thoroughly. Thus, users use BESSiE at their own risk.
